# The epidemiology of drowning in low- and middle-income countries: a systematic review

**DOI:** 10.1186/s12889-017-4239-2

**Published:** 2017-05-08

**Authors:** Matthew D. Tyler, David B. Richards, Casper Reske-Nielsen, Omeed Saghafi, Erica A. Morse, Robert Carey, Gabrielle A. Jacquet

**Affiliations:** 1 Boston Medical Center, Boston, USA; 2 Denver Health Medical Center, Denver, USA; 3 Boston University School of Medicine, Boston, USA

**Keywords:** Drowning, Injury, Epidemiology, Low and middle-income countries, Systematic review, Drowning prevention, Public health

## Abstract

**Background:**

According to the World Health Organization, drowning is the 3rd leading cause of unintentional injury-related deaths worldwide, accounting for 370,000 annual deaths and 7% of all injury-related deaths. Low- and middle-income countries are the most affected, accounting for 91% of unintentional drowning deaths.

**Methods:**

The authors performed a systematic review of literature indexed in EMBASE, PubMed, Web of Science, Cochrane Library, and Traumatology journals formerly indexed in PubMed in January 2014 and again in September 2016. Abstracts were limited to human studies in English, conducted in low- and middle-income countries, and containing quantitative data on drowning epidemiology.

**Results:**

A total of 62 articles met inclusion criteria. The majority of articles originate from Asia (56%) and Africa (26%). Risk factors for drowning included young age (<17–20 years old), male gender (75% vs. 25% female), rural environment (84% vs. 16% urban), occurring in the daytime (95% vs. 5% night time), lack of adult supervision (76% vs. 18% supervised), and limited swimming ability (86% vs. 10% with swimming ability). There was almost equal risk of drowning in a small body of water versus a large body of water (42% ponds, ditches, streams, wells; 46% lakes, rivers, sea, ocean).

**Conclusion:**

Drowning is a significant cause of injury-related deaths, especially in LMICs. Young males who are unsupervised in rural areas and have limited formal swimming instruction are at greatest risk of drowning in small bodies of water around their homes. Preventative strategies include covering wells and cisterns, fencing off ditches and small ponds, establishing community daycares, providing formal swimming lessons, and increasing awareness of the risks of drowning.

**Electronic supplementary material:**

The online version of this article (doi:10.1186/s12889-017-4239-2) contains supplementary material, which is available to authorized users.

## Background

Drowning is a major cause of morbidity and mortality worldwide, predominately affecting low- and middle-income countries (LMICs). According to the World Health Organization (WHO), drowning accounted for an estimated 372,000 deaths in 2012, with 91% of these deaths occurring in LMICs [1]. Children (1–18 years of age) [2] are especially susceptible with over 450 children drowning each day worldwide and thousands suffering debilitating injuries, including brain injury, as a result of drowning events [3]. Child injury surveys by the United Nations Children’s Fund (UNICEF) and The Alliance for Safe Children (TASC) indicate that drowning is the leading cause of childhood death in Asia [4]. The full impact of drowning deaths and disability due to drowning are grossly underreported, as many LMICs have limited resources to collect data [4]. In high-income countries (HICs), drowning risk factors include male gender, less than 14 years of age [5, 6], risky behavior including alcohol use [5, 7], low income [8], rural areas [4], and lack of supervision [5]. In this literature review, thousands of articles originating from LMICs were reviewed in order to highlight the populations at greatest risk of drowning worldwide and circumstances in which drowning occurs.

Understanding the epidemiology of drowning injuries is fundamental in directing preventative efforts. Many studies originating from HICs have proposed preventative measures to curb drowning deaths [9, 10]. HICs have made great strides in primary and secondary prevention due to sustained epidemiological research, improved information gathering systems, strict legislation, public health advocacy, and social marketing strategies. Recommendations include increasing public awareness and education, increasing supervision, and erecting fences around pool areas [9–12]. HIC interventions, however, are not universally applicable to LMICs as interventions specific to income level and region are necessary to account for varying epidemiologic, demographic, and cultural factors as well as differences in resources. In LMICs, issues such as the large burden of drowning, lack of epidemiological data, and limited public health infrastructure and outreach programs create further difficulties in implementing cost-effective methods of prevention [4, 13, 14]. This paper seeks to make recommendations for preventing drowning in LMICs based on the demographics of individuals at greatest risk of drowning and the reported epidemiology of drowning.

## Methods

**Fig. 1:**
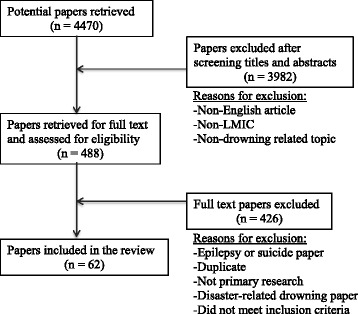
Search strategy

We performed a systematic review of the literature indexed in PubMed, EMBASE, Web of Science, Global Health, and the Cochrane Library database in January 2014 and again in September 2016. The Preferred Reporting Items for Systematic Reviews and Meta-Analyses (PRISMA) guidelines were followed to ensure a high-quality review. Search terms used were “drowning” or “near drowning” or “immersion” or “swimming/adverse effects” or “swimming/education.” The authors also hand-searched the Traumatology journals, which were in the National Library of Medicine catalog no longer indexed in PubMed. The initial search returned 4470 articles, 3982 of which were excluded after review of titles and abstracts. Upon primary review of the remaining 488 articles, 426 were excluded by two reviewers (with conflicts resolved by a third) using the following criteria: non-human subjects; not in English; not a complete manuscript (e.g. only an abstract, poster, presentation, lecture, letter, or short communication); not originating from a LMIC – as defined by the World Bank classification method (which utilizes Gross National Income as the comparative marker) as of January 2014; [15] not containing primary data on drowning incidents; focused on drowning related to suicide, epilepsy, or disaster events. Only English-language papers that included quantitative data on drowning epidemiology of humans in LMICs were included in this review. Data was then extracted from the remaining 62 manuscripts identified. Figure 1 provides details on the search strategy. Papers from 1984 to 2015 were included.

### Data collection and processing

Studies were reviewed for information pertaining to the occurrence of drowning. Specific data was collected from the studies, including: study population, source of data, age, gender, rural versus urban, location (outside versus inside, type of body of water), time of day, swimming ability, and presence of adult supervision.

These data were extracted from each paper into a Microsoft Excel spreadsheet and tabulated to identify trends. The data were then synthesized into a narrative. Two authors performed data extraction independently; a third author reviewed any ambiguous studies. A formal meta-analysis was not performed due to the diverse methods and definitions used in the studies.

## Results

**Fig. 2:**
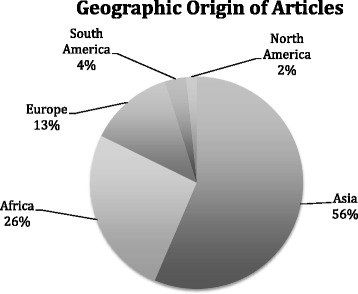
Geographic origin of articles

The search produced 4470 titles of interest, of which 488 were selected for full text review (Fig. 1). Finally, 62 empirical studies were identified as meeting all inclusion criteria. All 62 articles and their epidemiologic data provided are included in Additional file 1. Sources of data included autopsy and medico-legal records (22/62, 35%), databases (21/62, 34%), surveys and interviews (11/62, 18%), hospital records (5/62, 8%), media reports (2/62, 3%), and ambulance records (1/62, 2%). The majority of articles originated in Asia (35/62, 56%) and Africa (16/62, 26%); all regional origins are shown in Fig. 2 *.* Additional file 1 also includes drowning incidence totals for each epidemiological category as defined in this literature review: age, gender, location, setting, supervision, swimming ability, and time of day.

**Fig. 3:**
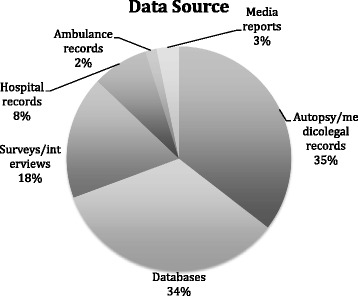
Sources of data

The countries with the highest numbers of articles were Bangladesh (*n* = 11, 18%) and South Africa (*n* = 11, 18%). Sources of data are shown in Fig. 3 and include predominantly autopsy/medico-legal records (*n* = 22, 35%).

## Discussion

In this systematic review, we analyzed 62 articles that met inclusion criteria in providing primary data on the epidemiology of drowning victims in LMICs. We examined characteristics of who was drowning and under what circumstances they were drowning. The majority of the data originated from Southeast Asia, Iran, and South Africa, which exemplifies that drowning is a global public health issue. After reviewing the final 62 articles, several trends were noted: young males with no swimming ability drowned with more frequency in small bodies of water close to home, in the daytime, and without adult supervision.

### Age

A total of 52 out of 62 articles contained primary data on the age of drowning victims and documented a total of 57,278 drowning events. The conclusions that can be drawn from this data are unfortunately limited due to two factors, 1) lack of consistency between articles in the age ranges they chose to report their data and 2) one article [16] contained an overwhelming majority of the data points with a reported 40,604 drowning incidents. Some articles only included young children <5 or <14 years old, others covered drowning deaths ranging from infants to the elderly. Several articles organized age by decades (0–10 years old, 11–20 years old, 21–30 years old, etc.), while others used relatively random age ranges (0–4 years old, 5–14 years old, 15–29 years old, 30–50 years old, etc.). To organize this data, we noted trends in the articles and identified several age ranges that could be used: <4–5 years old, <12–15 year old, >12–15 year old, <17–20 year old, and >17–20 year old. Nineteen articles included data specifically on victims less than the age of 17–20 years old (4192 drowning events in <17–20 year old age group and 3702 events in >20 years old) [17–34]. Twenty articles included data specifically on drowning events in children <12–15 years old (17,610 drowning events in children <12–15 years old; 29,600 drowning victims >12–15 years old) [16, 32, 35–54], and thirteen articles contained data on deaths in toddlers and infants <4–5 years old (2053 deaths in <4–5 year olds and 118 deaths in >4–5 year olds) [13, 55–66].

Due to the inconsistency in reported age ranges in the above articles, specifics cannot be provided on percentage of drowning deaths in each age group. The data is also skewed by the enormous drowning study described above, which reports a large number of drowning incidents in individuals >15 years old. However, this article does report a higher rate of drowning events per 100,000 people in 0–4 year olds and 5–14 year olds. The majority of the remaining articles also contain data that support an increased frequency of drowning deaths in younger individuals (<17–20 years old). Several articles provided primary data only on infants and toddlers <4–5 years old, which further supports the conclusion that young children are at greatest risk of drowning in LMICs.

### Gender

Males are at greater risk of drowning than females and accounted for 45,240 events (75%) from 50 out of 62 articles compared to 15,295 female events (25%) [13, 16–28, 30–33, 35–39, 41, 42, 44, 46, 48, 52–55, 57, 59–74]. Males generally exhibit riskier behavior than their female counterparts and therefore expose themselves to more dangerous situations when around bodies of water [75]. Additionally, young males are more frequently employed on boats and in water-centric jobs [76, 77]. Lastly, in many cultures, boys are given more freedom outside the home and frequently bathe in the ponds and streams, while girls are given less freedom to roam outside and restricted to bathing inside [57].

### Rural versus urban settings

Many of the countries included in this study have large urban centers and vast surrounding rural areas. Eight articles contained primary data comparing drowning deaths in rural and urban communities [32, 39, 59, 62, 63, 65, 73, 76]. There were a total of 4931 drowning victims in these eight articles: 4159 people drowned in a rural setting (84%), while 772 people drowned in an urban setting (16%). Seven of these articles originated from southeast Asian countries known for their extensive waterways and natural bodies of water: China (*n* = 3), India (*n* = 2), Pakistan (*n* = 1), and Bangladesh (*n* = 1). Rural areas in LMICs pose a significantly greater risk than urban areas to potential drowning victims, especially children at play.

### Body of water

Several articles further defined the demographics of drowning victims by the size or type of body of water they drowned in. They generally characterized the body of water as small (e.g., cisterns, wells, streams, ditches, ponds), large (e.g., river, lake, harbor, dam, ocean), indoor, or swimming pool. A total of 16 articles accounting for 4453 drowning victims contain primary data on the location and body of water. A total of 2047 (46%) drowning events occurred in large bodies of water, 1852 (42%) in small bodies of water, 326 (7%) unknown, 119 (3%) in swimming pools, and 109 (2%) indoor [18–20, 28, 30, 32, 37, 39, 41, 43, 57, 58, 64, 71, 78, 79].

The data was skewed by one particular article, which focused primarily on drowning incidents of tourists in the northern part of Iran [30]. The data was collected from an area around a beach resort and heavily favored ocean drownings (1042 in large bodies of water and 52 in small bodies of water). Excluding this article, the majority of drowning incidents occur in small bodies of water.

Cisterns and wells are common in LMICs and used as a source of water in small, rural communities. They are generally close to the home and are frequently uncovered. Streams and ponds are also common geographic features in rural communities and can be a setting of play for children. Not surprisingly, these small bodies of water are the most frequent location of drowning events. Of note, there was a relatively low number of drowning deaths in swimming pools, compared to high-income countries, in which a significant number of drowning incidents occur in pools [8, 80, 81]. Swimming pools are less common in low-income countries, especially in rural regions [53].

### Time of day

Eleven articles contained primary data on the time of day that drowning events occurred. [21, 25, 26, 29, 30, 38, 53, 56, 57, 61, 78]. However, six of these articles’ data were not reported in a way that could be concurrently analyzed with data from other articles [26, 30, 38, 53, 56, 78]. For instance, one article reported the time of the drowning event as AM, PM, evening, and night [26]. Five articles could be used for final analysis as they had overlapping ranges: daytime starting between 6:00–9:00 and ending between 17:00–18:00 [21, 25, 29, 57, 61]. A total of 1803 drowning events were recorded: 1714 in the daytime (95%) and 89 in the nighttime (5%). An overwhelming majority of drowning incidents occurred in the daytime, which is not surprising as this is when people are outside their homes and at greater risk for drowning in a body of water.

### Swimming ability

Three articles recorded data on whether or not the drowning victim had the ability to swim, defined as having prior swimming lessons or swimming ability identified by family or friends [29, 39, 53]. There were a total of 296 incidents recorded: 254 (86%) in which drowning victims did not have any swimming ability, 29 (10%) in which they had some swimming ability, and 13 (4%) in which they had unknown swimming ability. This is a small sample size, however several studies have shown a benefit to providing children with swimming lessons to reduce the incidence of drowning [53, 61, 82]. Unfortunately, swimming lessons are not prioritized in many LMICs, due in part to the fact that drowning is a grossly under recognized health hazard for children and also due to financial restrictions [8, 61].

### Supervision

Seven articles provided data on the level of supervision provided for each drowning incident [29, 35, 39, 53, 56, 57, 78]. In general, an individual >18 years old was considered adequate adult supervision while someone <18 years old was not considered to be an adult supervisor. Therefore, these articles only examine drowning amongst children. A total of 1804 drownings were recorded: 1376 (76%) of these were unsupervised, 319 (18%) were supervised, and 109 (6%) had unknown supervision. Adult supervision clearly reduces the incidence of drowning events, however, in many of the communities involved in these studies, the adult at home, generally the mother, is often busy with other children and household chores [29, 57, 61, 78]. As a result, they are unable to keep watch over their children at all times and it is during these moments of distraction that children are at greatest risk of drowning.

### Limitations

A major limitation of this systematic review was inconsistent data collection between articles, especially in terms of age ranges. The ‘Utstein style’ [83] is a recommended data recording system for drowning incidents and includes data points such as age, location of the drowning, time of day, event witnesses, CPR initiation, and EMS / ED assessment. A more robust assessment of post-drowning resuscitation could have been discussed if the ‘Utstein style’ had been followed by the included articles. Additionally, a more organized age distribution could have been included in this review if there was uniformity in the data collection. Additional limitations include the exclusion of articles primarily recording drowning due to suicide and epileptic events, articles not written in English, and disaster related drowning events. Therefore, despite clear patterns among the studies reviewed, the incidence of drowning is likely underreported overall.

## Conclusion

Drowning is a significant cause of injury-related deaths, especially in LMICs. Understanding drowning risk factors aids in implementation of effective preventative strategies. Young children should receive swimming instruction and communities should implement daycares to ensure constant adult supervision, especially in the daytime. Additionally, small ponds and irrigation ditches should be encircled with fences or drained, and cisterns/wells covered by grates to prevent children from falling into them. Finally, programs to educate parents on the risks of drowning should be implemented and could help reduce the childhood drowning rate by 40% [84]. These preventative strategies are especially important in rural areas where the risks of drowning are higher. The WHO in their Global Drowning Report also supports many of the recommendations included in this systematic review. Additional means of reducing the threat of drowning included in the Drowning Report and not captured in this review include timely rescue and resuscitation efforts by bystanders (CPR), signage and designation of hazardous water bodies, and comprehensive boating regulations and enforcement. This systematic review was limited by the inconsistent method of recording data between articles; therefore LMICs collecting primary data on drowning victims should follow a supported incidence template, such as the “Utstein template”. Further research is necessary to explore the effectiveness of preventative strategies in low and middle-income countries.

## Additional files

Additional file 1: Articles Selected for Inclusion and Demographics of Drowning Deaths. (XLSX 20 kb)

## Abbreviations

HICs: High-income countries
LMICs: Low- and middle-income countries
TASC: The Alliance for Safe Children
UNICEF: United Nations Children’s Fund
WHO: World Health Organization
